# Sleep duration and Framingham´s cardiovascular risk score: results from the Hoveyzeh Cohort Study (HCS)

**DOI:** 10.1186/s12872-023-03611-2

**Published:** 2023-11-20

**Authors:** Bahman Cheraghian, Habib Heybar, Nader Saki, Maedeh Raeisizadeh, Seyed Jalal Hashemi, Saeid Bitaraf

**Affiliations:** 1 Department of Biostatistics and Epidemiology, School of Public Health, Ahvaz Jundishapur University of Medical Sciences, Ahvaz, Iran; 2 Atherosclerosis Research Center, Ahvaz Jundishapur University of Medical Sciences, Ahvaz, Iran; 3 Hearing Research Center, Clinical Sciences Research Institute, Ahvaz Jundishapur University of Medical Sciences, Ahvaz, Iran; 4 Department of Biostatistics and Epidemiology, School of Public Health, Clinical Sciences Research Institute, Ahvaz Jundishapur University of Medical Sciences, Ahvaz, Iran; 5 Department of Internal Medicine, School of Medicine, Alimentary Tract Research Center, Ahvaz Jundishapur University of Medical Sciences, Ahvaz, Iran; 6 Clinical Sciences Research Institute, Ahvaz Jundishapur University of Medical Sciences, Ahvaz, Iran

**Keywords:** Cardiovascular diseases, Sleep duration, Iran, Cohort studies

## Abstract

**Background:**

Cardiovascular diseases (CVDs) are the leading causes of global deaths. So, it is necessary to know the possible risk factors for each region. We aimed to assess the relationship between the risk of CVDs and sleep duration in the Iranian Arab population.

**Methods:**

In this cross-sectional study, data from 8362 participants aged between 35 and 70 from the Hoveyzeh Cohort Study enrollment phase between 2016 and 2018 was analyzed. This study evaluated the association between ten-year CVD risk using the Framingham risk score and sleep duration. Self-reported sleep duration was categorized as short duration (equal to or less than 5 h), standard duration (6–8 h), or prolonged duration (equal to or greater than 9 h). Multivariable logistic regression to adjust for conventional CVD risks was used.

**Results:**

Among the assessed participants, 4892 (58.50%) were female, and the mean age was 47.93 (9.01). The average sleep duration was 7.59 (1.56) hours. The low, intermediate-to-high, and high CVD risk individuals were 6461 (77.27%), 1185 (14.17%), and 716 (8.56%), respectively. There was a significant relationship between prolonged sleep duration and lower odds of intermediate-to-high CVD risk in males (OR 0.66, 95% CI: 0.49–0.87, *P*-value: 0.004).

**Conclusion:**

The results of our study showed that longer sleep duration has a protective association with the intermediate to high risk of cardiovascular diseases in males. However, long and short sleep durations have no significant relationship with females’ risk of cardiovascular disease.

## Background

Cardiovascular diseases (CVDs), especially ischemic heart disease (IHD) and stroke, are the leading causes of global deaths and important contributors to disability [1]. In 2020, about 523 million people were estimated to have at least one form of CVD, and around 19 million deaths were attributed to CVDs; this accounted for approximately 32% of all global deaths [2]. Moreover, CVDs are responsible for 46% of the total deaths and 20%–23% of the disease burden in Iran [3].

Several known cardiovascular risk factors are modifiable, such as a sedentary lifestyle, obesity, smoking, hypertension, and hyperlipidemia [4]. Using these risk factors, some risk scores, such as Framingham, SCORE, PROCAM, and Globorisk, have been devised to predict the risk of cardiovascular diseases [5]. The Framingham Risk Score (FRS) was presented to predict a 10-year CVD risk incidence [6]. Besides the mentioned CVD risk factors, some other suggested risk factors may be associated with FRS: fatty liver disease, metabolic syndrome, Alzheimer's disease, and sleep duration [7]. There is controversy about the association of CVD risk with sleep duration [8]. Some studies showed an association only between short sleep duration and increased CVD risk but not for long sleep duration, and some indicate an inverse relationship [8, 9]. Some studies demonstrated the association between elevated CVD risk and short and long sleep duration; some rejected these [10, 11]. Several studies have shown a U-shaped relationship between FRS and sleep duration. The U-shaped relationship indicates that short and long sleep durations have a positive and significant relationship with cardiovascular diseases [12]. To the best of our knowledge, the relationship between sleep length and FRS has not been investigated in the Iranian Arab population. For this purpose, this study aimed to assess the association of sleep duration with the Framingham risk score of CVDs in the population aged between 35 and 70 of the Hoveyzeh Cohort Study (HCS), a cohort study on the Iranian Arab population.

## Methods

### Study design and participants

In this cross-sectional study, the baseline data of the Hoveyzeh Cohort Study (HCS), collected between May 2016 and August 2018, was used. This study is a branch of the Prospective Epidemiological Research Studies in Iran (PERSIAN) cohort study in the southwest of Iran, which has been run on the Iranian Arab population [13, 14]. Residents of Hoveyzeh, aged 35 to 70, were included in the study. After excluding patients with proven cardiovascular disease (1483) and participants who were sleeping pill users (164), the data from 8362 participants was analyzed.

### Measurements

A checklist containing demographic information, sleep duration, and risk factors for CVD, including hypertension, dyslipidemia, diabetes mellitus, active smoking, BMI, and physical activity, was used to collect the data.

Systolic blood pressure (SBP) and diastolic blood pressure (DBP) were measured twice (10 min apart) on each arm using Riester sphygmomanometers following standard protocols. SBP ≥ 140 mmHg, DBP ≥ 90 mmHg, previous clinical diagnosis of HTN, use of blood pressure-lowering drugs, or self-reported diagnosis of HTN were all indicators of HTN [15]. Increased triglyceride (> 200 mg/dL), LDL (> 160 mg/dL), and total cholesterol levels (≥ 240 mg/dL) or decreased HDL levels (< 40 mg/dL) were all considered to be dyslipidemia [16]. Diabetes was characterized by a fasting blood sugar level of 126 mg/dL or higher, the use of glucose-lowering medications, or a self-reported diagnosis of the disease. Individuals who had smoked over 100 cigarettes during their lifetime were considered active smokers. BMI was calculated by dividing a person's weight in kilograms by their height in meters squared (kg/m2). A BMI of 18.5 or less was considered underweight; 18.5 to 24.9 was considered healthy; 25.0 to 29.9 was considered overweight; and 30 or more was considered obese. The metabolic equivalent of task (MET Index), which expresses the intensity of physical activities, was determined to assess the participant's level of physical activity. MET is the ratio of a person's working metabolism to their resting metabolic rate. We used the daily physical activity scale to calculate MET for each participant's activities throughout the 24 h [13].

### Calculation of sleep duration

Self-reported sleep duration was categorized as short duration (equal to or less than 5 h), standard duration (6–8 h), or prolonged duration (equal to or greater than 9 h) [12].

### Calculation of the Framingham risk score

The Framingham risk score was calculated regarding gender and age using total cholesterol, high-density lipoprotein (HDL) cholesterol, systolic blood pressure, hypertension treatment, smoking status, and diabetes mellitus [6]. Since some variables for calculating FRS are used in different manners in females and males, and originally, this index was calculated separately in two gender groups [6], FRS was calculated and analyzed separately in this study. In participants without CVD history, FRS is categorized into low CVD risk (less than 10%), intermediate CVD risk (from 10 to 20%), and high CVD risk (more than 20%) groups [12].

### Sample size

This study was conducted in the context of the enrollment phase of HCS. Participants in HCS were 10009 and were selected using the census method. After excluding patients with proven cardiovascular disease (1483) and sleeping pill users (164), the data from 8362 participants was analyzed. However, a power analysis was performed to ensure the adequacy of the sample size. A power higher than 0.8 indicates a sufficient sample size [17].

### Statistical analysis

Study data are shown as frequencies in percentage, means ± standard deviation, and medians (interquartile range). The data were analyzed using the chi-square test, analysis of variances, Kruskal–Wallis, and logistic regression. The proportional odds assumption must be met for ordinal regression. The assumption was violated in our data, so conventional logistic regression was used.

In this study, we used two logistic regression models. The first model was a crude model to examine the crude association between sleep duration and FRS. The second model was adjusted, in which age, dyslipidemia, body mass index, and physical activity were controlled for this relationship. Several confounders for the relationship between sleep duration and cardiovascular disease are found in the literature. A number of them were not available in the present study and since the outcome variable of this study was the Framingham risk score and this index is a composite index including variables such as diabetes and hypertension, in the adjusted analysis, these variables were not controlled. In these models, the effect of sleep length on the difference between high and low CVD risk and intermediate and low CVD risk has been measured.

*P*-values less than 0.05 were considered statistically significant. To conduct all analyses, we used STATA version 14.

## Results

Participants’ baseline characteristics are demonstrated in Table 1 according to sleep duration. Short, standard, and prolonged sleep durations comprised 740 (8.85%), 5299 (63.37%), and 2323 (27.78%) of the study participants. Females and older people were more prevalent in the prolonged sleep duration group. Dyslipidemia and obesity (obese and overweight) were more prevalent in subjects of the short sleep group, and diabetes mellitus, smoking, and hypertension were more prevalent in participants of the prolonged sleep group.

**Table 1:** Baseline characteristics (Sociodemographic, clinical and laboratory characteristics) by sleep duration categories

**Characteristics**		**Sleep duration**			
		**< = 5 h**	**6–8 h**	**> = 9 h**	***p*-value**
		***N* = 740**	***N* = 5299**	***N* = 2323**	
**Age**^**a**^		46.47 (± 8.11)	47.62 (± 8.75)	49.10 (± 9.70)	< 0.001
**Gender**^**b**^	Male	503 (68.00%)	2330 (44.00%)	637 (27.40%)	< 0.001
**Hypertension**^**b**^	Yes	129 (17.40%)	1081 (20.40%)	524 (22.60%)	0.007
**Dyslipidemia**^**b**^	Yes	342 (46.20%)	2062 (38.90%)	863 (37.20%)	< 0.001
**Diabetes**^**b**^	Yes	132 (17.80%)	1028 (19.40%)	483 (20.80%)	0.16
**Smoking**^**b**^	Yes	212 (28.06%)	1081 (20.40%)	376 (16.20%)	< 0.001
**BMI**^**b**^	Underweight	5 (0.68%)	73 (1.38%)	50 (2.15%)	< 0.001
	Normal	159 (21.49%)	1202 (22.68%)	576(24.80%)	
	Overweight	321 (43.38%)	2017 (38.06%)	796 (34.27%)	
	Obese	255 (34.46%)	2007 (37.88%)	901 (38.79%)	
***Physical activity(MET)***^***c***^		*38.21 (34.90–42.57)*	*37.05 (34.25–40.60)*	*35 (32.30–37.50)*	*< 0.001*

*BMI*  body mass index, *MET*  metabolic equivalent of task

^a^mean (± standard deviation)

^b^number (%) and

^c^median (IQR)

The prevalence of FRS categories at every sleeping level is shown in Table 2. The most prevalent sleep category was standard sleep duration (67.15% [95% CI: 65.58%–68.71%] in males and 60.70% [95% CI: 59.32%–62.06%] in females). Standard sleep duration was also the most prevalent in every FRS category. There was a gradient in the frequency of FRS levels in sleep duration categories. In the short and standard sleep duration groups, the percentage of participants decreased when FRS increased.

**Table 2:** Prevalence of FRS categories in sleep duration levels

FRS	Sleep Duration in female		
	< = 5 h	6–8 h	> = 9 h
< 10%	217(5[4.4, 5.7])	2662(61.2[59.8, 62.7])	1464(33.8[32.3, 35.1])
10–20%	15(3.6[2.1, 6.0])	228(56.3[51.4, 61.3])	161(41.1[35.0, 44.8])
20% ≤	5(3.4[1.1, 7.8])	79(54.4[46.0, 62.8])	61(42.2[33.9, 50.5])
FRS	Sleep Duration in Male		
	< = 5 h	6–8 h	> = 9 h
< 10%	339(16.0[14.5, 17.6])	1440(68[65.9, 69.9])	339(16.0[14.5, 17.6])
10–20%	106(13.6[11.2, 16.2])	534(68.4[64.9, 71.6])	141(18.0[15.4, 20.9])
20% ≤	58(10.2[8.7, 12.9])	356(62.3[58.2, 66.3])	157(27.4[23.8, 31.3])

Values are numbers (prevalence [95%CI])

*FRS* Framingham risk score

However, the results were the opposite in terms of prolonged sleep duration. An unadjusted logistic regression has shown a statistically significant relationship between high-risk FRS and sleep duration in the male group. This relationship for short sleep duration was negative (OR 0.69, 95% CI: 0.51–0.94, *P*-value: 0.02), and for long sleep duration, it was positive (OR 1.87, 95% CI: 1.50–2.34, *P*-value < 0.001). Univariable logistic regression has not shown any association between sleep duration and FRS levels in the intermediate-to-high-risk male group. A crude logistic regression had only demonstrated an association between prolonged sleep duration and intermediate-to-high risk (OR 1.28, 95% CI: 1.03–1.59, *P*-value: 0.02) in the female group (Table 3). An adjusted model containing age, dyslipidemia, body mass index, and physical activity has shown a relationship between prolonged sleep duration and intermediate-to-high risk of cardiovascular disease only in males (OR 0.66, 95% CI: 0.49–0.87, *P*-value: 0.004) (Table 3). Therefore, sleep duration is associated with 10-year intermediate-to-high-risk cardiovascular diseases in the only males with prolonged sleep duration in the population of the Hoveyzeh cohort.

**Table 3:** Crude and adjusted odds ratio for FRS by sleep duration categories using logistic regression

		Intermediate-to-high risk			High risk		
Gender	Variables	OR	95% CI	*p*_value	OR	95% CI	*p*_value
Male	Univariable model						
	sleep < = 5	0.84	0.66–1.07	0.16	0.69	0.51–0.94	0.02
	sleep > = 9	1.12	0.90–1.40	0.31	1.87	1.50–2.34	< 0.001
	sleep 6–8	Reference group					
	Adjusted model						
	sleep < = 5	1.01	0.75–1.34	0.97	0.91	0.60–1.39	0.66
	sleep > = 9	0.66	0.49–0.87	0.004	0.75	0.53–1.07	0.11
	sleep 6–8	Reference group					
Female	Univariable model						
	sleep < = 5	0.81	0.47–1.38	0.44	0.78	0.31–1.94	0.59
	sleep > = 9	1.28	1.03–1.59	0.02	1.40	0.99–1.97	0.06
	sleep 6–8	Reference group					
	Adjusted model						
	sleep < = 5	1.07	0.59–1.96	0.82	1.15	0.43–3.06	0.77
	sleep > = 9	0.95	0.74–1.24	0.74	0.95	0.64–1.41	0.82
	sleep 6–8	Reference group					

Logistic models are adjusted for age, dyslipidemia, body mass index and physical activity

*CI* Confidence interval

Finally, a power analysis showed that the power of the present study with a sample size of 8362 and considering the probability of type Ι error equal to 0.05 is more than 0.95.

## Discussion

The main results of this study showed that long sleep duration was significantly associated with intermediate-to-high CVD risk in the male population of the Hoveyzeh cohort study. In this study, sleep duration did not have a significant relationship with the risk of cardiovascular diseases in females.

Although a 10-year follow-up study from NHANES I showed no significant relationship between coronary heart disease risk and sleep duration [9], most research demonstrates an association between short sleep duration and an increased risk of CVDs. On the other hand, the influence of long sleep duration is inconsistent [18]. Some studies have shown that short and slightly less prolonged sleep durations increase the risk of chronic diseases [19]. The results of some studies illustrated a U-shaped association between sleep duration (short and long) and CVD risk score [19, 20]. This inconsistency in different studies on the relationship between CVDs and sleep duration can be caused by different sample sizes and particular population profiles and compositions. Additionally, certain unmeasured or unaccounted confounding variables, such as depression, may affect the relationship between sleep duration and CVDs [21].

There is no clear explanation for the gender difference in the relationship between sleep duration and cardiovascular disease. Some different mechanisms may influence the impact of sleep on CVD in males and females in different ways [22]. Some studies have shown that sleep-related problems may impact females more strongly. It can be due to hormonal differences, more insomnia, and worse sleep quality in females [23, 24].

In line with our study, Farjam et al. have shown that longer sleep duration had a protective relationship with CVD, MI, and CHD, but the observed association was insignificant [25]. Frajam's study is based on the data of the Fasa cohort, another branch of the PERSIAN cohort study. Everyday habits among Iranian ethnic groups can cause this similarity between the two studies. In the same direction, according to findings from a meta-analysis, longer sleep length could improve the cardiometabolic condition [26]. These findings can be explained by recent studies showing that longer sleep duration, especially for those with chronic sleep deprivation, can lower the risk of obesity and enhance cardiovascular health [21].

Differences in demographic, cultural, and habitual factors may influence sleep duration and quality and their consequences [25]. A study in the Korean population and age groups older than 18 revealed that long and short sleep durations are related to increased cardiovascular disease [12]. In contrast, our study was done on the Iranian Arab population aged 35 to 70. A similar study was conducted on the Persian Fasa cohort of the Fars ethnicity in Iran. This study has shown that short sleep duration is a risk factor for cardiovascular diseases. However, no significant relationship was observed between long sleep and the Framingham risk score [25]. The differences between the current study and the Fasa cohort study can be attributed to differences between ethnic groups in sleep length, which can lead to different health conditions. The present study was done in a community with rural behaviors that may differ from those of the urban population. A multi-ethnic cohort study showed an association between sleep duration and some cardiovascular risks like obesity and diabetes mellitus only in special ethnic groups [27]. Another study has demonstrated an effect modifier role for ethnicity in the relationship between sleep duration and CVD risks. Mentioned study has shown a strong association between sleep length and obesity in African Americans and a strong association between sleep duration and hypertension in some other ethnicities [28]. Although the cause of this discovery is not entirely known, it may be partially attributed to variations in dietary habits and lifestyle factors, which may vary throughout ethnic groups and may modify the relationship between sleep duration and CVD risk factors [27].

In 2019, cardiovascular disorders were responsible for more than 30% of fatalities worldwide and 40% of deaths in Iran [29]. A study evaluated Iran's non-communicable diseases (NCDs) prevention program, indicating that the economic cost of NCDs to the Iranian economy amounted to 5% of the country’s yearly Gross Domestic Product (GDP). With an increase of 29.9% over the previous ten years, ischemic heart disorders were the most common. In addition, the majority of the major risk factors for deaths and disabilities in Iran were either intermediate-risk factors, such as high blood pressure, high body mass index, high fasting plasma glucose, and abnormal lipid profiles, or behavioral risk factors for NCDs, such as tobacco use and dietary risks [30]. On the other hand, a cost-effectiveness analysis of Iran's primary prevention program (IraPEN) for cardiovascular diseases revealed that the most cost-effective strategies were preventive therapies that target high-risk individuals [29]. For this purpose, it is recommended that higher quality and longer sleep duration, especially for those with chronic sleep deprivation, be considered a CVD prevention factor for the population mentioned above, besides other factors affecting CVD.

### Strengths and limitations

One of our study's strengths, which helped us produce more accurate estimates, was the sample size of our population-based cohort study. Second, the employment of the questioners who have received proper training and the presence of several tiers of supervisors. Third, the study of the Arab population’s ethnicity and way of life among Iranian Arabs was essentially the same as that of nearby nations, particularly those from the south of Kuwait and Iraq. Our study limitations included the fact that, due to the cross-sectional nature of this study, causality cannot be inferred from the study's findings. Using sleep duration without considering sleep quality may lead to confounding conclusions. Our study does not comprise the measurement of apnea, a recognized risk factor for cardiovascular disease [31]. Because of disease severity diversity, using binary variables as confounders may not control confounding variables completely.

Cardiovascular diseases include coronary heart disease (CHD), myocardial infarction (MI), and stroke. On the other hand, FRS predicts the total risk of cardiovascular diseases. Therefore, in this study, it was impossible to investigate the relationship between the future risks of each subgroup of cardiovascular diseases and sleep length. Finally, the present study was conducted on the 35 to 70-year-old population, and its results cannot be generalized to other age groups.

## Conclusion

According to our research findings, longer sleep duration was associated with a lower risk of cardiovascular diseases in men. This finding can be considered by those responsible for making policies and programs that aim to reduce the risk of cardiovascular diseases in the mentioned population. It is recommended that this relationship be investigated in prospective cohort research to better understand it.

## Data Availability

The datasets used and/or analyzed during the current study are available from the corresponding author upon reasonable request.
